# Report of Two Cases of Brain Tumor1Read before the Buffalo Academy of Medicine, April 13 1909.

**Published:** 1909-08

**Authors:** Henry P. Frost

**Affiliations:** Buffalo, N. Y.


### Report of two Cases of Brain Tumor[1]

By HENRY P. FROST, M. D., Buffalo, N. Y.

TUMORS of the brain, while not excessively rare, are infrequent enough in the experience of most of us to justify the presentation here for your inspection, of two interesting specimens obtained recently from autopsies at the Buffalo State Hospital. They illustrate very well the marked difference in origin, structure and mode of growth, of the two most common varieties of brain tumor, endothelial sarcoma and glioma. The first of these an essentially benign growth, originating from the endothelial cells of lymph spaces, springs usually from the dura, most often at the base of the brain, and growing slowly, displaces the brain tissue, and hollows out for itself a bed without actually invading the brain. It is of firm, fibrous consistency, and can be shelled out from its resting place without much difficulty.

The glioma, on the other hand, a more malignant type, has its origin from neuroglia tissue in the depth of the brain and grows more rapidly, infiltrating and destroying the brain substance. It is softer in consistency, especially in the center, resembling, it may be, as in the case shown tonight, a thrombotic softening, but with somewhat firmer margins, more or less hemorrhagic, not sharply defined but merging into the surrounding healthy tissue. The first mentioned variety is naturally, except for its frequent inaccessibility of site, much the more suitable for operation. The brief clinical histories presented herewith will illustrate the widely differing clinical course of the two types

1. Read before the Buffalo Academy of Medicine, April 13 1909.

no less clearly than the tumors themselves show their dissimilarity in situation and make up.

Case I.—Sarcoma—E. F., female, age 44, a widow with five healthy children. Admitted to Buffalo State Hospital, May 25, 1907. Family history, negative. Father living, age 75; mother died of cirrhosis of the liver. Patient has seven brothers and sisters, all healthy. Personal history: previous health good. She never had any serious illness, nor suffered any injury; habits temperate; no lues, normal menopause within the year. She was of average intelligence and was an energetic, active woman.

*History of present illness.*—For about seven years she had frequently complained of severe headache, the pain running up the back of the head and across the top. Three years before her commitment her physician, Dr. Alexander Allen, examined her eyes and found optic atrophy in both, but vision was not seriously impaired until two years later when her sight failed rapidly, first in the right eye, and in six months she was totally blind. Before this the headaches had increased in severity, and she suffered from dizziness on suddenly assuming the erect posture or making any quick movement. The patient was seen at this time by Dr. William C. Krauss in consultation, who diagnosticated an inoperable brain tumor affecting the orbital lobes at the base of the brain. At times, after an exacerbation of headache, she was very dull for a day or two, with some flightiness and incoherence of speech. Later there was permanent impairment of the mind, marked by hebetude, failure of memory, fabrication of imaginary experiences, and inability to grasp her actual situation and surroundings, together with some ill defined delusions of grandeur and wealth. Later still and after complete loss of vision, she developed hallucinations of sight and hearing. She saw imaginary figures and heard singing which she did not recognise as unreal. For perhaps two years before we saw her she was subject to "spells like nervous chills," lasting five or ten minutes. In some of these attacks her knees would give way, and she occasionally fell and was unconscious or nearly so. Four months before admission she had a severe general convulsion followed by stupor for twenty-four hours. For a long time she had complained much of feeling cold. An early symptom was pain at the root of the nose, to relieve which an operation was performed several years ago. There had been no vomiting. Patient had been thoroughly treated with mercury and iodide of potassium without benefit. She gained forty pounds in weight during a year of inactivity preceding commitment to hospital.

On admission patient presented the mental symptoms already noted, and this condition underwent no important change while she lived, except steady progression. The most striking feature throughout was drowsiness, interrupted by periods of aimless restlessness and confusion of ideas. She always yawned a great deal. I shall detail only her subjective complaints and the neurological symptoms. She complained of a feeling of weakness and vertigo, whether lying or standing; a sensation of turning to the right; headache, principally in the forehead, and worse on the right side; "neuralgia" of right side of face; pain in the thighs "zig-zag like a saw," and in the back. She often rubbed the right thigh and leg, and seemed at times to suffer more pain there than in the head.

*Physical examination.*—Patient had a smoothed out face and wore a dull, stolid expression. There was slight bulging of the eyes more marked in the right, no ptosis; slight external strabismus of right eye. The eyes could be turned in all directions, but not so far to the left as to the right; convergence was impossible; pupils equal, dilated, immobile. No vision; ophthalmoscopic picture: outline of discs lost, marked swelling of discs; veins enormously enlarged and tortuous, with scattered hemorrhages through the retina; more marked congestion in right.

Hearing normal on both sides; noise like singing heard, "deep in the head." Taste normal. Smell abolished in both nostrils. Articulation, phonation and deglutition normal. Cutaneous sensibility normal, also sense of position and stereognostic sense. No motor paralysis or localised weakness; tongue protruded in median line; no tremor, except in tongue; no ataxia; station and gait very good. Superficial reflexes normal; tendon reflexes moderately increased, symmetrical; organic reflexes deranged. Urine and feces passed involuntarily; eventually automatic masturbation; infrequently vomiting.

While in the hospital she had at irregular intervals, between thirty and forty convulsions, none of them severe. In the seizures she frothed at the mouth and breathed stertorously and was sometimes rigid in the upper extremities, but in many of them she had no definite spasm, except in the respiratory muscles. Once the eyes were noticed to deviate to the left; aside from this the convulsions furnished no aid for localisation. In a final series, terminating in death, the attacks were more severe, with opisthotonos, universal rigidity and clonic spasms. Death occurred January 26, 1909, one year and eight months after admission and at least six years after the first appearance of symptoms.

Our diagnosis was a tumor in the frontal region, probably on the right side. The signs which indicated this location were: the severity of the mental involvement, especially of the higher intellectual functions, the absence of motor paralysis or localised convulsions, also the absence of cerebellar symptoms, such as ataxia, and the integrity of the cranial nerves having their origin from the posterior portion of the brain stem. The sensory disorder (neuralgia) in the distribution of the right trifacial, was the principal reason for assigning the neoplasm to the right side, and there was also in favor of this assumption the right sided symptom of damage to the motor oculi, as well as the doubtful evidence afforded by the convulsive deviation of the eyes to the left. The very striking symptom of anosmia, showed that the lesion, if it did not involve the left frontal lobe, at least reached to the middle line, and was therefore inaccessible for operation.

*Autopsy.*—The calvarium was moderately thick. There were no unusual adhesions between the skull and dura. The dura was thin, tense and bulging. Upon incising it the brain presented every appearance of extreme compression; the pia was practically bloodless, and the brain looked very pale; it filled the dura as snugly as possible and was perfectly dry—not a drop of fluid was seen. The convolutions were flattened and squeezed together so that the sulci were seen as mere linear markings on a plane surface.

In the median line at the base of the brain was found a large solid tumor, firmly adherent to the crista galli and cribriform plates of the ethmoid bone, from which point it extended backward and upward, displacing and distorting both frontal lobes. Its surface was rough and warty; its consistency firm and hard, and the size, measured in situ, 7.5 x 5 c.m. The sella turcica was enlarged and contained a cystic pituitary body. The tips of the temporal lobes were firmly adherent to the base of the skull; and there were minute punched out spots scattered over the surface of the brain, particularly in the temporal and frontal lobes. The brain with tumor attached weighed 1,380 gms.

PATHOLOGICAL REPORT BY DR. JOSEPH B. BETTS.

*Microscopic examination.*—The tumor microscopically is a typical enthothelioma (endothelial sarcoma.) The endothelial cells are arranged somewhat spirally, forming whorls, but without the formation of pearls, such as are seen in epitheliomata. The growth is not markedly vascular, and no areas of necrosis are found.

CASE II.—A. H., male, age 63, married; habits temperate; no previous illness. He was an intelligent man, engaged in active business up to two months before the end of his life, and until then showing no signs of brain disease, except some change in disposition for several months, which was attributed to business worries.

Patient was admitted to Buffalo State Hospital February 14, 1909, with a history of memory defect and occasional confusion, since the beginning of January. At family prayers he could not say the Lord's prayer as usual. He spoke very little, usually only "yes" or "no," saluted when addressed instead of answering, seemed depressed and absorbed in thought, made peculiar mistakes, *e. g.*, after promising his grandchild a present he handed her his railroad ticket; he urinated in a tea kettle. He seemed to comprehend what was said to him, and obeyed directions.

He failed rapidly in physical condition as well as mentally. When received at the hospital he was unable to walk or stand alone; was very dull and stuporous, scarcely moved in bed, had incontinence of urine and feces. If questioned sharpely he would open his eyes and look duly at examiner and perhaps shake his head; several times he attempted to say a few words but he could not be understood.

*Physical Examination.*—The right side of the face was smoothed out, drooped a little, and was sucked in and out by the breathing; the right arm was not moved voluntarily as much as the left and seemed weaker—very feeble grip in both. When assisted to walk the right foot was dragged. Both knee jerks were diminished; no Babinski. Cutaneous sensibility everywhere preserved, but dulled. Heart slightly enlarged, action good, no murmur; arterial tension moderate; some sclerosis of radials and temporals. Pupils equally contracted, slight reaction to light; vision apparently good, no hemianopsia, slight arcus senilis. Urine contained albumen, granular casts and sugar. Tumor was not suspected; a diagnosis of brain softening from arterial degeneration was made, and uremia was regarded as a probable additional factor in the stupor. Patient died in stupor without development of any further symptoms ten days after admission.

*Autopsy.*—The dura was tense and upon opening it there was scarcely any c.s. fluid. The brain was very large, weighing 1,785 gms. Pia somewhat congested, but thin and not adherent. A bulging was noticeable in the left frontal lobe. In this region a small area of hemorrhagic appearance was slightly elevated above the surface. Section at this point disclosed a large cavity, with ragged hemorrhagic looking wall, containing a grey pulpy necrotic material. The appearance was very similar to brain softening or old hemorrhage, but the tissue surrounding the broken down area was thick and firm enough to suggest tumor. The cerebral vessels were not sclerosed, and the aorta was practically normal.

The spleen was large and filled with small, fairly firm nodules, slightly paler than the spleen tissue; they varied in size from a small olive to a pigeon's egg. There was a large "nutmeg" liver. The pancreas was congested and near the tail there was an ecchymotic spot.

The right adrenal was normal; the left contained two nodules the size of an olive, firm, reddish grey in color, easily shelled out from the gland. The kidneys were enlarged and congested.

PATHOLOGICAL REPORT BY DR. JOSEPH B. BETTS.

*Microscopic examination.*—Vertical sections were taken from the periphery of the tumor, including the adjacent cerebral cortex and extending through the shell of the tumor into the softened central portion.

*Microscopically.*—There is no sharp line of demarcation between the brain tissue and the tumor mass, but as we approach the edge of the tumor, swollen neuroglia cells become more and more numerous, and the tissue here is much more vascular than normal cortex. The growth is made up of neuroglia cells, of the large spider type, many with greatly swollen cell bodies.

Multinuclear cells are numerous, and there is occasionally a true giant cell somewhat resembling those found in gummata and tubercles. An occasional mitosis is found in the younger portions of the tumor, indicating perhaps a rather rapid growth.

The tumor is extremely vascular. The vessels dilated and thin walled. Many contain thrombi and are surrounded by extravasations of blood. The softened necrotic areas in the tumor are accounted for by these thromboses.

If we include a cyst of the thyroid, this patient furnished tumors in four of his organs. The growth in the adrenal body proved to be a hypernephroma, from which the smaller ones in the spleen were evidently metastases. There was no connection between these and the cerebral tumor, which was of an entirely different type as already described.

BUFFALO STATE HOSPITAL.

